# A highly sensitive flexible capacitive pressure sensor with hierarchical pyramid micro-structured PDMS-based dielectric layer for health monitoring

**DOI:** 10.3389/fbioe.2023.1303142

**Published:** 2023-11-09

**Authors:** Luyu Lv, Tianxiang Liu, Ting Jiang, Jiamin Li, Jie Zhang, Qihui Zhou, Rajendra Dhakal, Xiao Li, Yuanyue Li, Zhao Yao

**Affiliations:** ^1^ Heart Center, Qingdao Hiser Hospital Affiliated of Qingdao University (Qingdao Traditional Chinese Medicine Hospital), Qingdao University, Qingdao, China; ^2^ College of Electronics and Information, Qingdao University, Qingdao, China; ^3^ School of Rehabilitation Sciences and Engineering, University of Health and Rehabilitation Sciences, Qingdao, China; ^4^ Department of Computer Science and Engineering, Sejong University, Seoul, Republic of Korea; ^5^ Hisense Visual Technology Co., Ltd., Qingdao, China

**Keywords:** PDMS, capacitive pressure sensor, hierarchical pyramid microstructure, high sensing performance, physiological signals monitoring

## Abstract

Herein, a flexible pressure sensor with high sensitivity was created using a dielectric layer featuring a hierarchical pyramid microstructure, both in simulation and fabrication. The capacitive pressure sensor comprises a hierarchically arranged dielectric layer made of polydimethylsiloxane (PDMS) with pyramid microstructures, positioned between copper electrodes at the top and bottom. The achievement of superior sensing performance is highly contingent upon the thickness of the dielectric layer, as indicated by both empirical findings and finite-element analysis. Specifically, the capacitive pressure sensor, featuring a dielectric layer thickness of 0.5 mm, exhibits a remarkable sensitivity of 0.77 kPa^-1^ within the pressure range below 1 kPa. It also demonstrates an impressive response time of 55 ms and recovery time of 42 ms, along with a low detection limit of 8 Pa. Furthermore, this sensor showcases exceptional stability and reproducibility with up to 1,000 cycles. Considering its exceptional achievements, the pressure sensor has been effectively utilized for monitoring physiological signals, sign language gestures, and vertical mechanical force exerted on objects. Additionally, a 5 × 5 sensor array was fabricated to accurately and precisely map the shape and position of objects. The pressure sensor with advanced performance shows broad potential in electronic skin applications.

## Introduction

The flexible pressure sensor serves as a core component for electronic skin (Guo et al., 2022; Zheng et al., 2022; Zhu et al., 2022), enabling the emulation of human skin’s sensing mechanism and exhibiting promising potential in wearable device applications (Lin et al., 2020; Lee et al., 2021; Lyu et al., 2021), smart prosthetics (Tian et al., 2019; Khoshmanesh et al., 2021), health monitoring (Rocha et al., 2021; Wang et al., 2022; Han et al., 2022), and body motion detection (Xiong et al., 2020; Zhu et al., 2021), etc. The classification of pressure sensors can be based on five distinct sensing mechanisms: piezoresistivity (Ding et al., 2020; Wang et al., 2022b; Ji et al., 2022), capacitance (Li et al., 2020; Qin et al., 2021; Wang et al., 2022c; Duan et al., 2022), piezoelectricity (Wang et al., 2021; Luo et al., 2021; Yi et al., 2022), field-effect transistors (Shi et al., 2020; Cheng et al., 2023), and triboelectricity (Wang et al., 2021; Liu et al., 2021; Xu et al., 2022). Among them, the capacitive pressure sensor has garnered substantial research for the superiority of outstanding structural stability, rapid response time, minimal power consumption, and a compact circuit design. Similar to the conventional parallel-plate capacitor, capacitive pressure sensors typically employ a “sandwich” structure, comprising a dielectric layer sandwiched between two electrodes positioned at the top and bottom. Its capacitance (*C*) is determined by the dielectric layer’s permittivity (*ε*), the effective area between two electrodes (*A*), and the separation distance of plate electrodes (*d*). Generally, the vertical force on the capacitive pressure sensor can induce variations in the dielectric layer thickness, leading to corresponding changes in the capacitance measurement. Therefore, it's vital to select a suitable material and optimize the structure configuration for the dielectric layer. Furthermore, the preparation of capacitive pressure sensors with controllable morphology on a large scale poses a significant challenge in achieving high performance.

The commonly utilized materials for fabricating the flexible dielectric layer include elastomers like polydimethylsiloxane (PDMS) (Wan et al., 2018; Hwang et al., 2021), polystyrene (Tian et al., 2020; Su et al., 2021), polyurethane (PU) (Seyedin et al., 2020; Zhu et al., 2020), etc. Among them, PDMS has emerged as the dominant choice for the dielectric layer owing to its reduced Young’s modulus, enhanced thermal stability and improved chemical stability. Moreover, the implementation of the micro-structured designs on the dielectric layer, such as micro-pyramid (Yang et al., 2019; Li et al., 2020; Zhang et al., 2021; Tao et al., 2022), micro-porous (Li et al., 2020; He et al., 2020), micro-sphere (Jung et al., 2020; Xiong et al., 2020), micro-pillar (Luo et al., 2019; Cao et al., 2020), micro-wrinkles (Zeng et al., 2019; Tang et al., 2021), etc., has demonstrated its efficacy in enhancing sensor performance. Generally, the realization of the microstructure is based on the template replication approach, which consists of soft-lithography and hard-lithography. Soft-lithography technique exploits bionic micro-patterns to make microstructures via directly copying the morphology of natural substances. Wan et al. developed an exceptional sensitivity (1.2 kPa^-1^) flexible tactile sensor by utilizing lotus leaf as template to obtain bionic microtowers array structure m-PDMS substrate (Wan et al., 2018). Jian and colleagues developed pressure sensors that exhibit exceptional performance, featuring an impressive sensitivity of 19.8 kPa^-1^ and an incredibly low detection threshold of 0.6 Pa. These sensors were constructed using a highly conductive active film combined with a bionic hierarchical microstructured PDMS substrate, which was replicated from the leaves of E. aureum plant species (Jian et al., 2017). However, it is difficult for large-scale fabrication. In addition, it suffers from an inherent flaw where the microstructure’s shape, dimension, and spacing remain unalterable. Therefore, it is not feasible to fabricate structured micro-patterns with a predetermined form and dimension. The hard-lithography is an effective approach that can tackle the above problems. The hard-lithography depends on photolithography technique and wet etching to fabricate a patterned template that can be transferred to a flexible polymer material. Luo et al. have successfully designed a capacitive sensor utilizing a tilted pillar array dielectric layer, showcasing exceptional sensitivity of 0.42 kPa^-1^ below 1 kPa. The dielectric layer was molded from the tilted micro-structured template made by photolithography (Luo et al., 2019). In the same way, Tao and colleagues produced a novel dielectric layer using ionic gels with pyramidal-shaped microstructured to obtain an unprecedented sensitivity of 41 kPa^-1^ (Tao et al., 2022). Thanks to the implementation of a porous pyramid dielectric layer, Yang’s group prepared an ultrahigh sensitive (44.5 kPa^-1^) capacitive pressure sensor, which was designed to be unaffected by strain and temperature (Yang et al., 2019). The manufacturing process has merits of high precision, controllable aspect ratio, and mass production. Therefore, the hard-lithography technique enables the production of a microstructure that not only facilitates the efficient manufacturing of pressure sensors with superior performance on a large scale but also fulfills the requirement for convenient alteration of the microstructure’s morphology.

In this research, an efficient hard-lithography technique was employed to fabricate an exceptional sensitivity capacitive pressure sensor. The sensor utilized copper foils for both the bottom and top electrodes, along with a PDMS dielectric layer that incorporates a hierarchical pyramid microstructure. The prepared flexible capacitive pressure sensor exhibits a remarkable sensitivity of 0.77 kPa^-1^ below 1 kPa. It also demonstrates an impressive response time of 55 ms and recovery time of 42 ms, along with a low detection limit of 8 Pa. Furthermore, this sensor showcases exceptional stability and reproducibility with up to 1,000 cycles. Moreover, the comparison was conducted to evaluate the impact of dielectric layer’s microstructure and thickness on capacitive pressure sensors’ sensitivity. Meanwhile, the sensing performance was further evaluated through finite element analysis (FEA) to investigate the impact of dielectric layer’s microstructure and thickness. The increased deformation of the sensor with microstructure and thinner dielectric layer under identical pressure is responsible for this phenomenon. The development of a 5 × 5 sensor array was undertaken to facilitate the identification of spatial pressure allocation exerted by various objects. Ultimately, the artificially created pressure sensor exhibits a vast array of potential applications, encompassing monitoring human biological signals, detecting body motion and vertical mechanical pressure.

## Experimental section

### Preparation process of a patterned silicon template

The patterned template was fabricated through the utilization of photolithography on <100> silicon wafers that were covered with a 300 nm thermally grown oxide layer. The processes involved in photolithography can be described follows (Ruth et al., 2020). Firstly, the silicon wafer underwent a cleaning process using acetone/IPA/DI water, followed by drying with N_2_ blowing. Secondly, the silicon wafer’s surface was spin-coated with the photoresist (KXN5735-L0 negative photoresist) at 500 rpm for 6 s and a subsequent coating at 3,000 rpm for 20 s. The photoresist film was subjected to a preliminary baking process at 100°C for 90 s and exposed by the UV aligner. The wafer backed again at 120°C for 90 s. Thirdly, the photoresist film was developed by TMAH (2.38%) for 30 s and post-baked at 120°C for 3 min before wet etching. Next, the silicon oxide layer was etched by buffer oxide etchant (BOE) for 5 min. Eventually, the special hierarchical pyramid microstructure of the silicon template was fabricated by anisotropic etching using 5 M KOH solution for 5 h.

### Fabrication process of a pressure sensor with hierarchical pyramid microstructure

Firstly, the PDMS and the curing agent were meticulously mixed in a 10:1 weight ratio. Secondly, after a 30-min treatment in the vacuum chamber, the mixture’s bubbles were entirely eliminated. The mixture was introduced into the silicon template and subjected to spin-coating at a speed of 1,000 rpm, then cured at a temperature of 85°C for a duration of 1.5 h. It is important to mention that the spin-coating rate during the same period has an impact on the thickness of the dielectric layer. The dielectric layer was subsequently removed from the silicon template featuring a hierarchical pyramid microstructure. Ultimately, a flexible pressure sensor was developed by integrating a dielectric layer featuring hierarchical pyramid microstructures with copper foils as the upper and lower electrodes.

### Fabrication of a 5 × 5 sensor array

Five strips of copper foil with dimensions of 5 mm × 80 mm were applied to the polyimide (PI) tape in parallel at 5 mm intervals as the bottom electrode array. The identical procedure was employed to fabricate the top electrode array. The dielectric layer was fabricated using template replication technique, featuring a hierarchical pyramid microstructure. Once prepared, it was equally divided into 25 portions, each measuring 5 mm × 5 mm. The dielectric layer was securely adhered to the bottom electrode strips, while the top electrode strips were arranged orthogonally to form a 5 × 5 capacitive pressure sensor arrays (Ren et al., 2022).

### Measurement of sensing performance

The artificially created sensor was setup on the pressure gage (ZQ-990, Zhiqu) test platform, while its upper and lower electrodes were linked to the precision LCR meter (E4980, Keysight). The capacitive response was measured by applying varying force, utilizing a precision LCR meter with 100 kHz, 1 V bias. A USB connection was established between the LCR meter, ZQ-990, and a laptop for the purpose of post-processing and analyzing data. The surface morphologies of the patterned silicon template and the prepared PDMS film were scanned using field-emission scanning electron microscopy (FESEM, Hitachi S-4800, Japan) to obtain high-resolution images.

## Results and discussion

### Fabrication and characterization

**FIGURE 1:**
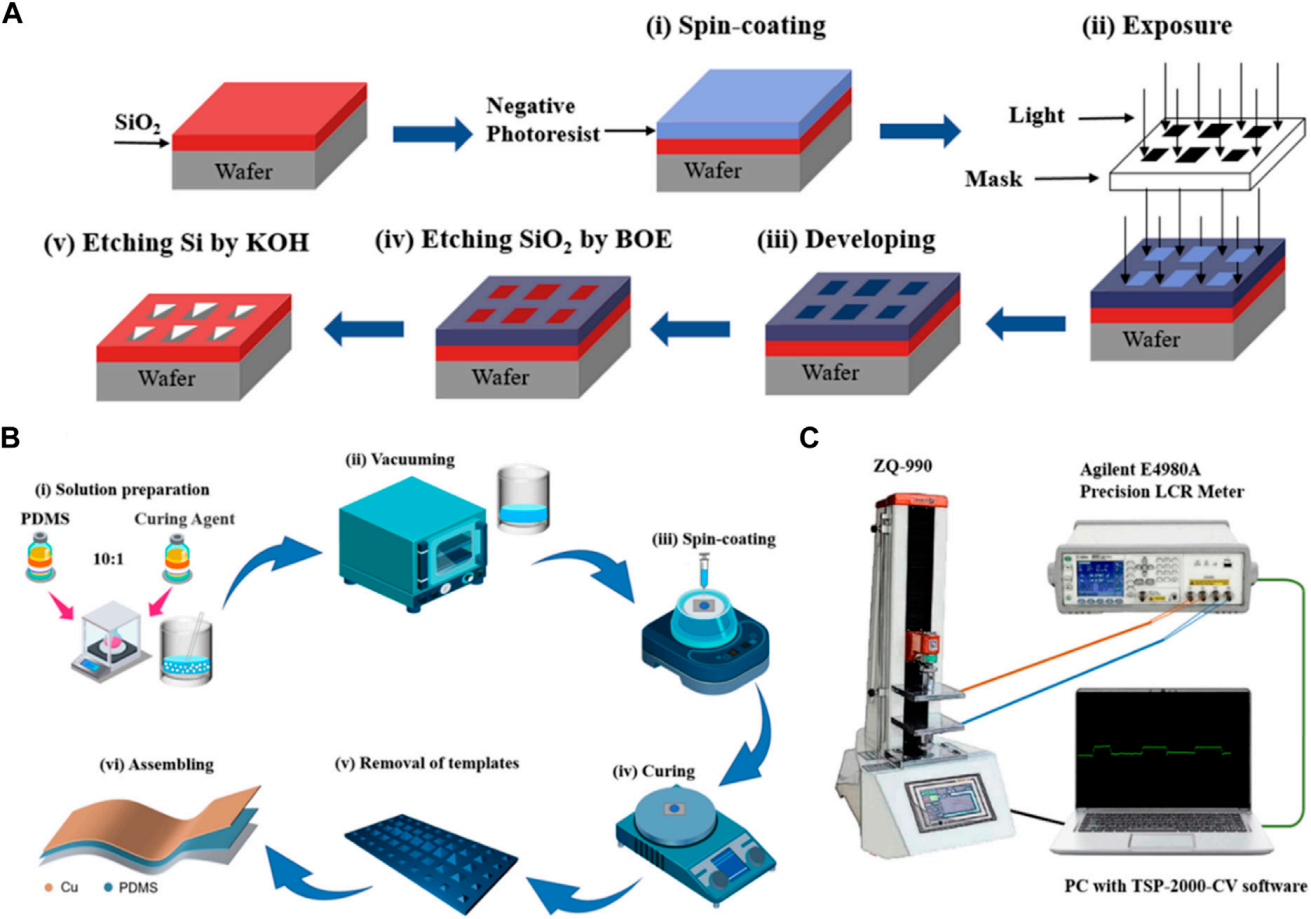
**(A)** Flowchart of the photolithography process for the patterned silicon template. **(B)** Schematic representation of the fabrication process for the flexible capacitive pressure sensor. **(C)** Experiment setup for the sensing performance measurement.

**FIGURE 2:**
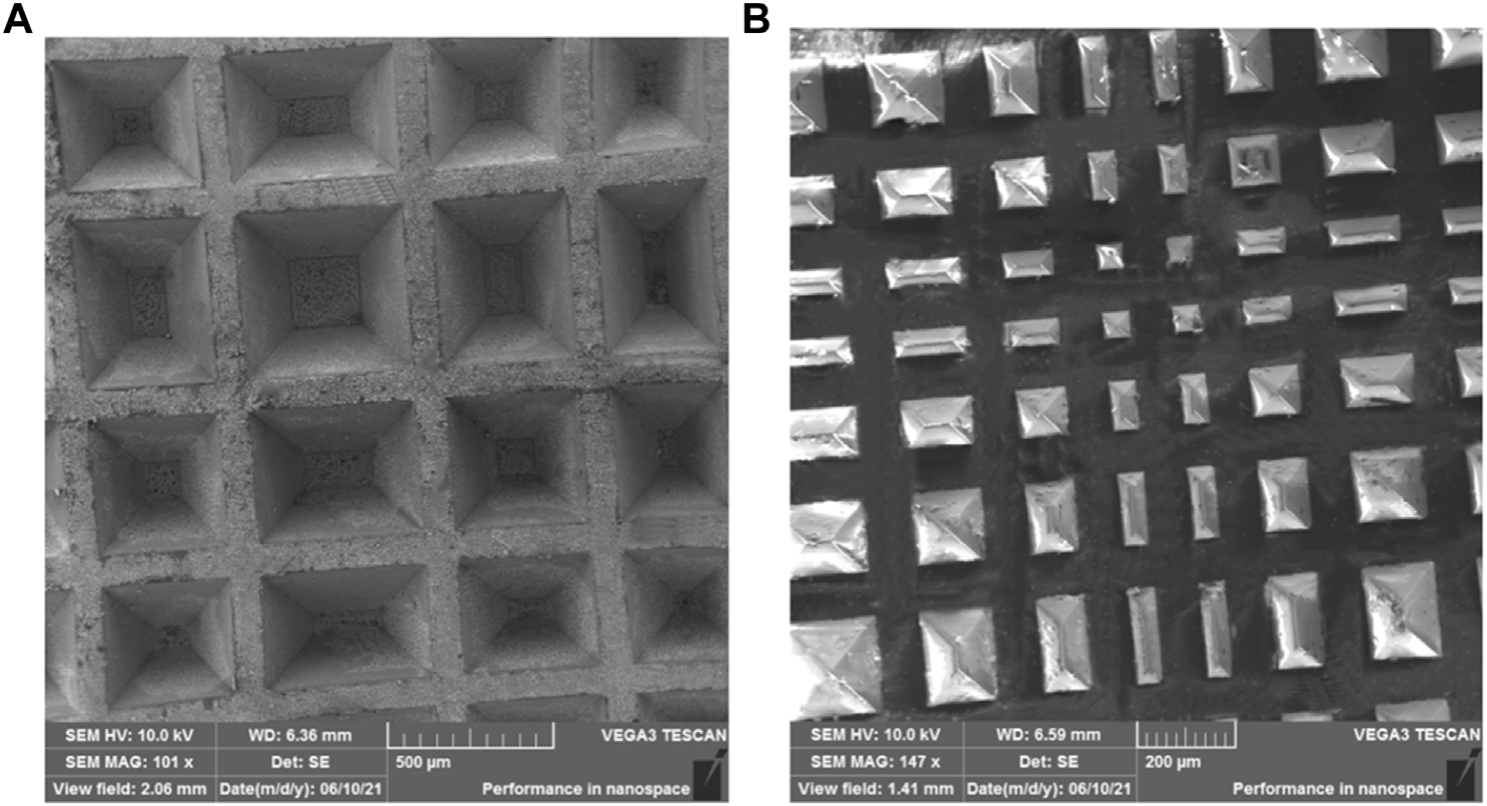
**(A)** SEM image of the patterned silicon template. **(B)** SEM image of the pyramid-structured PDMS film.

The flowchart of the photolithography process for the patterned silicon template are shown in Figure 1A. The fabrication process of the capacitive pressure sensor with the hierarchical pyramid microstructure dielectric layer is depicted in Figure 1B. The experiment setup for the sensing performance measurement is illustrated in Figure 1C. The Experimental Section provides a detailed account of the fabrication techniques employed and the methodology used for measuring the sensing performance. The SEM images of the patterned silicon template and the micro-structured PDMS film are depicted in Figure 2. The top view of the patterned silicon template reveals variations in microstructure size (Figure 2A), resulting in a visually striking hierarchical pyramid appearance. The distribution of these pyramids on the template exhibits a higher central peak and lower side peaks. The base of the pyramid is either a square or a rectangle in shape. The base of the pyramid exhibits a size variation ranging from approximately 40 μm–500 μm, while its height spans between 28 μm and 350 μm. After the templating process, the PDMS films display hierarchical pyramid microstructures as shown in Figure 2B.

### Pressure sensing performance of the flexible capacitive pressure sensor

The capacitive pressure sensor operates on the principle that its capacitive sensitive element converts the pressure signal into an electrical signal output that is directly proportional to the applied pressure. Similar to the conventional parallel-plate capacitor, the capacitive pressure sensor employs a “sandwich” structure, comprising a dielectric layer sandwiched between two electrodes positioned at the top and bottom (Li et al., 2018). Its capacitance *C* is determined by the equation:

$$C=\frac{\varepsilon_{0}\varepsilon_{r}A}{d}$$ (1)

where *ε*_0_ and *ε*_r_ denote the dielectric constants of the vacuum and the dielectric layer. When a perpendicular pressure is exerted on the sensor, there will be a change in *d*, resulting in a modification of *C*. Conversely, *A* undergoes alteration when subjected to shear force (Zhao et al., 2015).

By optimizing the microstructures of the dielectric layer (Cao et al., 2020; Tao et al., 2022), incorporating silver nanowires into the dielectric layer (Fu et al., 2022), or distributing nanoparticles on the surface of the dielectric layer, significant enhancements can be achieved in terms of sensitivity for capacitive pressure sensors (Kim et al., 2018). The sensitivity in the first case is attributed to a reduction in *d*, while in the latter case it is due to an increase in *ε*_r_. Incorporating microstructures into the dielectric layer significantly reduces its viscoelastic properties and greatly shortens both response and relaxation time. Based on the above discussion, a hierarchical pyramid microstructure dielectric layer has been designed. In comparison to other structures, this particular configuration enables greater deformation or higher change rate, resulting in rapid variations in capacitance and enhanced sensitivity.

**FIGURE 3:**
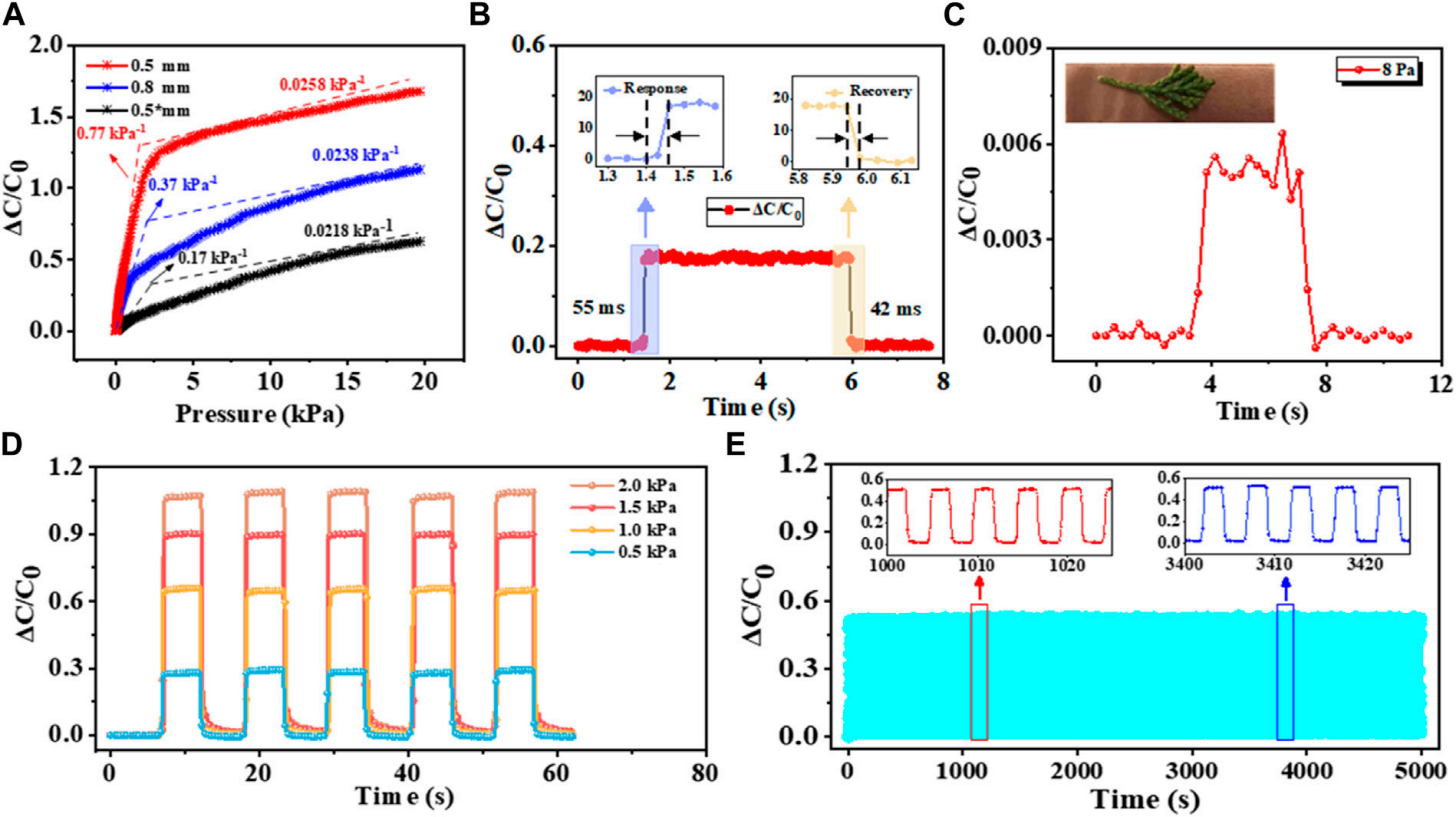
Sensing performance of the capacitive pressure sensor. **(A)** Sensitivity curves of capacitive pressure sensors based on the hierarchical pyramid microstructure dielectric layer with thicknesses of 0.5 mm (red) and 0.8 mm (blue), as well as the non-patterned dielectric layer with a thickness of 0.5 mm (black), respectively. **(B)** Real-time response under vertical pressure of ∼400 Pa (the insets show response and recovery time). **(C)** Capacitance response under a small pressure of ∼8 Pa. **(D)** Repeated real-time capacitance responses under 0.5, 1.0, 1.5, and 2.0 kPa. **(E)** 1,000 cycles for testing the stability of sensor under 0.8 kPa (the insets show the magnified view under different periods).

The initial estimation of capacitive pressure sensor’s fundamental sensing performance is conducted by applying vertical pressure. The evaluation of sensing performance heavily relies sensitivity (*S*), which is commonly characterized as (Niu et al., 2020):

$$S=\frac{\delta \left(\Delta C/C_{0}\right)}{\delta P}$$ (2)

where *C*_0_ denotes the capacitance at the beginning and *ΔC* represents difference in capacitance (*C*-*C*_*0*_), while *P* signifies the amount of vertical pressure exerted on the sensor. Based on the principle, the slope of the tangent of the pressure-capacitance curve reflects the level of sensitivity. As illustrated in Figure 3A, the variations in relative capacitance of the hierarchical pyramid microstructure sensor with the dielectric layer thickness of 0.5 mm was studied across a wide pressure range. To explore the impact of the dielectric layer’s microstructure and thickness of on sensitivity, additional sensors with two different types of dielectric layers were produced for comparison: a planar dielectric layer measuring 0.5 mm in thickness and a hierarchical pyramid microstructure dielectric layer measuring 0.8 mm in thickness. As shown in Figure 3A, the hierarchical pyramid microstructure pressure sensor with a dielectric layer thickness of 0.5 mm outperforms the other two instances. When the pressure is below 1 kPa, the hierarchical pyramid microstructure sensor with a dielectric layer thickness of 0.5 mm exhibits a sensitivity of 0.77 kPa^-1^, which significantly surpasses the sensitivity (0.37 kPa^-1^) of the sensor having a dielectric layer thickness of 0.8 mm. Conversely, the sensor featuring the planar dielectric layer demonstrates a comparatively lower sensitivity of 0.17 kPa^-1^. When the pressure exceeds 1 kPa, there is a reduction in sensitivity for the aforementioned three sensors to 0.0258 kPa^-1^, 0.0238 kPa^-1^, and 0.0218 kPa^-1^, respectively. The exceptional sensitivity can be primarily attributed to the distinctive micro-pyramid architecture, as evidenced by the information provided earlier. Moreover, the correlation between the sensitivity and dielectric layer thickness becomes more evident as the dielectric layer thickness decreases from 0.8 mm to 0.5 mm, highlighting the impact of varying dielectric layer thickness on pressure sensor’s sensitivity.

In addition to assessing sensitivity, an analysis on the response and recovery time measured by TSP-2000-CV was also performed. The sensor’s capacitance quickly stabilizes within a mere 55 ms (top-left inset) when subjecting an object to a load of approximately 0.4 kPa, showcasing its exceptional response time, as illustrated in Figure 3B. After the elimination of the applied force, there is a rapid decline in the sensor’s capacitance from its stable state to its initial value within a brief response period of 42 ms (top-right inset). It can be comparable to the human skin, typically ranging from 30 to 50 ms. Such remarkable accomplishments can be ascribed to the PDMS’s low viscoelasticity and the hierarchical pyramid microstructure. The capacitive pressure sensor is highly sensitive to detecting minute variations. With the assistance of the lightweight object (∼8 Pa), the apparent alteration in capacitance during the loading/unloading process can be visible in Figure 3C. Its repeatability and discrimination capability under five repetitive exerting/releasing cycles with different vertical pressure is further assessed. The results in Figure 3D demonstrate real-time monitoring. By applied 0.5, 1.0, 1.5, and 2.0 kPa, respectively, the relative capacitance variation exhibits notable fluctuations while maintaining consistent levels under identical pressure condition. This suggests super repeatability and the ability to discern varying degrees of pressure. Moreover, given the paramount importance of long-term stability in practical applications, 1,000 loading/unloading cycles at 0.8 kPa were conducted to ensure its robustness. As shown in Figure 3E, the uniform waveforms devoid of discernible fatigue indicate that the sensor possesses prominent stability and reproducibility.

**FIGURE 4:**
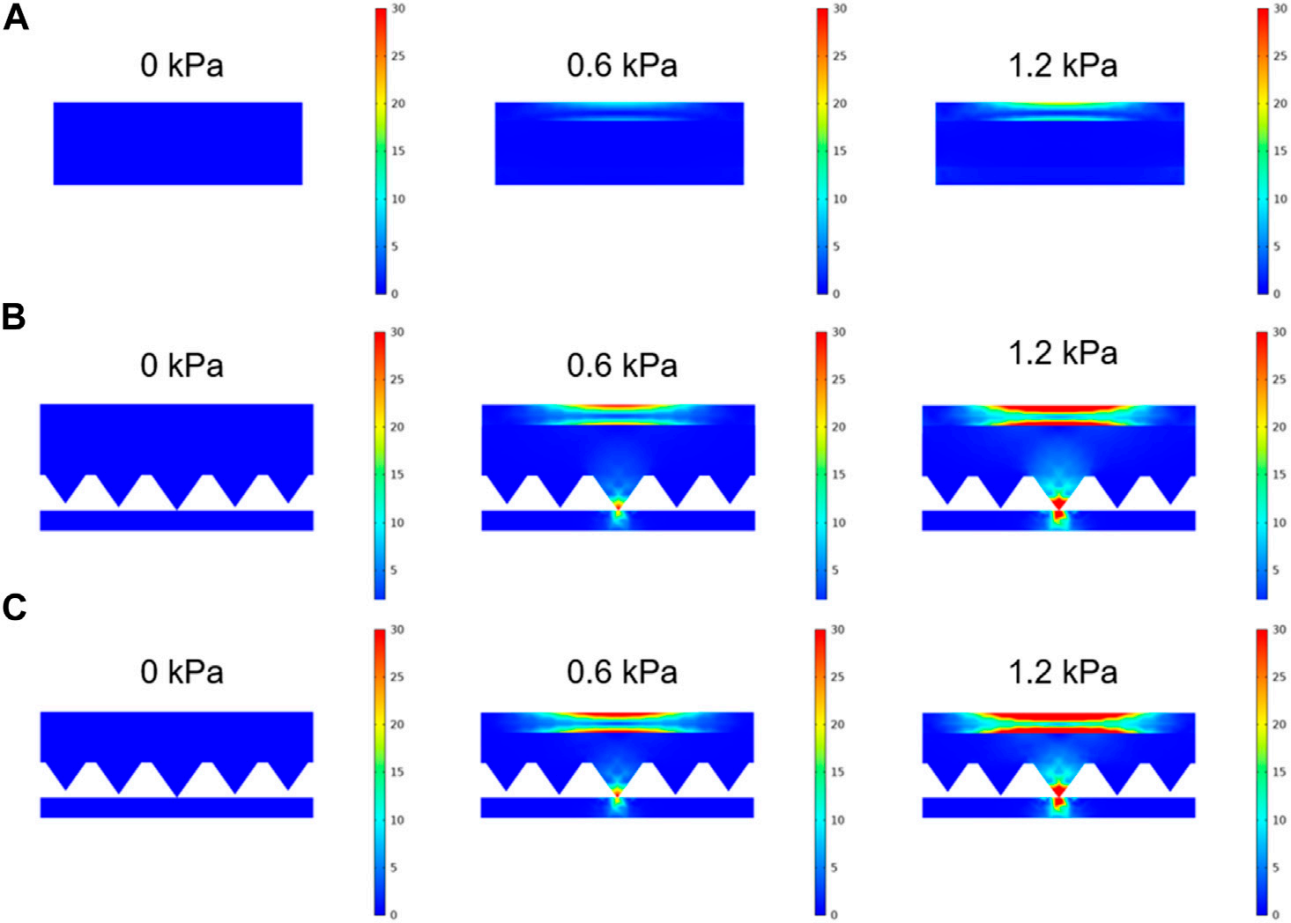
FEA simulation results of the pressure sensors. **(A)** Non-patterned sensor’s contact stress distribution with a dielectric layer thickness measuring 0.5 mm under 0, 0.6, and 1.2 kPa, respectively. **(B)** Hierarchical pyramid microstructure sensor’s contact stress distribution with a dielectric layer thickness of 0.8 mm under 0, 0.6, and 1.2 kPa, respectively. **(C)** Hierarchical pyramid microstructure sensor’s contact stress distribution with the dielectric layer thickness of 0.5 mm under 0, 0.6, and 1.2 kPa, respectively.

The sensing mechanism’s validation was extended through FEA to examine the impact of dielectric layer’s microstructure and thickness. The pressure sensors incorporated the dielectric layer configurations mentioned above were employed for comparative analysis. Figures 4A–C show the different stress distributions of the three sensors under 0, 0.6, and 1.2 kPa, respectively. As demonstrated in Figure 4A, under external vertical force, the whole contact surface of the planar sensor is deformed and the stress is distributed rather uniform. According to the parallel-plate capacitor definition, in this case, capacitance change is primarily influenced by the separation distance due to infrequent variations in the contact area. Therefore, the sensitivity is extremely low as a result of the minimal variation in the distance between adjacent planes. However, for the hierarchical pyramid microstructure pressure sensor (Figure 4B, C), the concentration of force on the microstructure is enhanced under identical pressure, resulting in increased deformation of the dielectric layer. That will increase the contact area and decrease the gap simultaneously, ultimately resulting in the improvement of the sensitivity. Moreover, when subjected to identical pressure, the thin sensor demonstrates a more extensive distribution of contact stress compared to the thick sensor. Consequently, this leads to an increase deformation. In general, the hierarchical pyramid microstructure pressure sensor with a thin dielectric layer causes the highest sensitivity, aligning well with the experimental findings.

### Wearable applications of flexible capacitive pressure sensor

**FIGURE 5:**
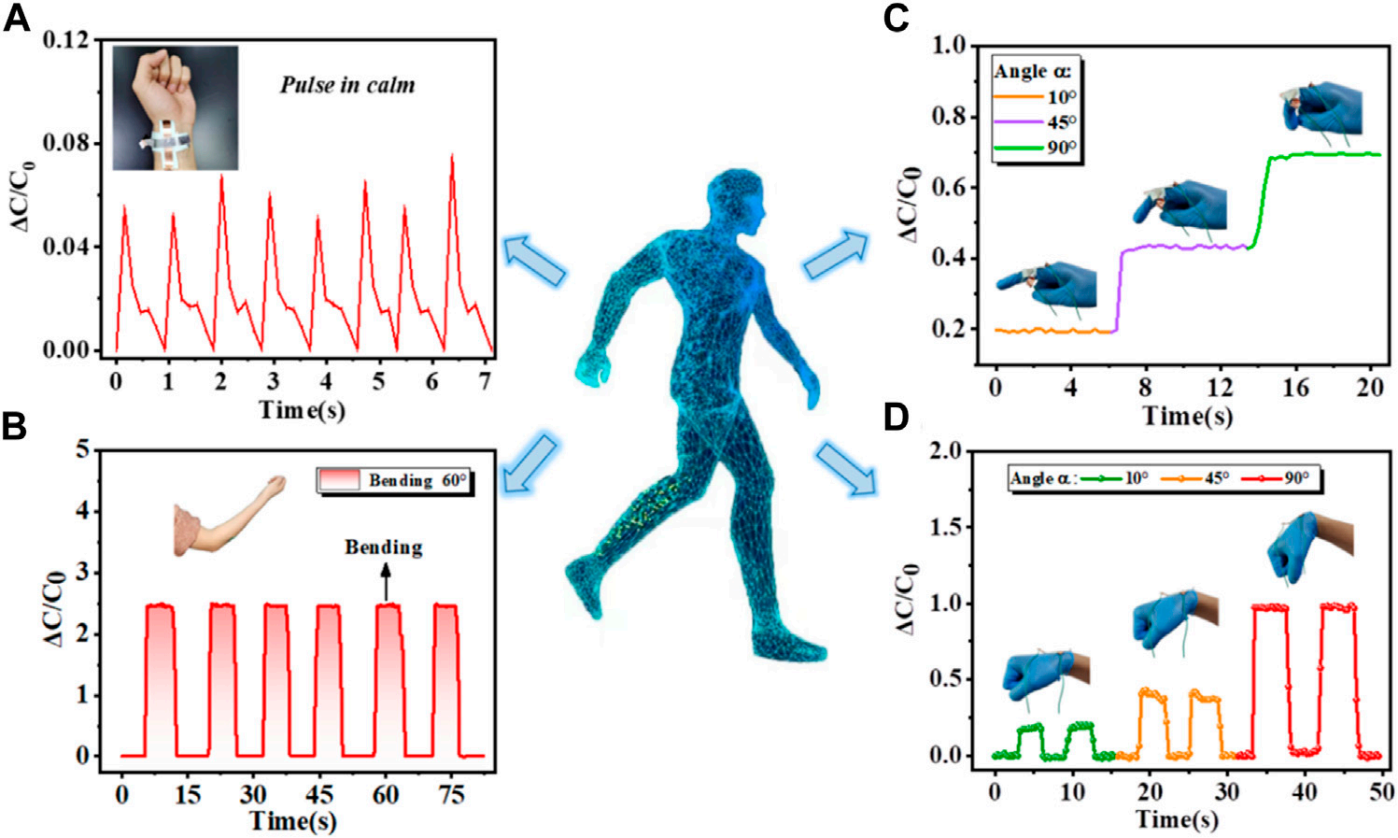
Various applications in recognition of physiological signals and human body motions. Real-time capacitance responses of different body positions including monitoring **(A)** wrist pulse under steady breath, **(B)** elbow with a bending angle of 60°, **(C)** finger, and **(D)** wrist with bending angles of 10°, 45°, and 90°, respectively.

A pressure-sensitive device was attached to various points on the human body, including the wrist, elbow, and finger. Its purpose was to monitor physiological signals and track bodily movements including pulse rate, bending of the elbow, flexing of fingers, and twisting of the wrist, respectively. The pulse signals are crucial physiological indicators of an individual’s health status. To identify wrist pulse, the pressure sensor has adhered to the participant’s wrist and the resulting signals were showcased in Figure 5A. The displayed data and replicable pulse patterns, with a frequency of approximately 1 Hz that closely resembles that of a human wrist pulse. Hence, the sensor encouraging prospects in the surveillance of feeble biomedical signals. Besides, capacitance responses of the elbow bending are shown in Figure 5B. When the elbow undergoes flexion from a state of relaxation to an angle of 60°, there is a rapid and significant increase in the relative change in capacitance by up to 250%, which then stabilizes. Conversely, when the elbow is extended, there is a prompt decrease in the relative capacitance change from 250% to 0%. Then volunteer repeats the above steps and the uniform waveforms are obtained. It is indicated that the sensor demonstrates a swift response/recovery time and excellent stability in the presence of external force. Finally, the sensor’s sensing performance is further assessed by placing it on the index finger and wrist while examining its response to various bending conditions. The initial position of the finger is set at a flexion angle of 10°, followed by subsequent adjustments to angles of 45° and 90°. It is evident from Figure 5C that the corresponding relative capacitance changes are ∼20%, ∼45%, and ∼70%, respectively. The test results manifest a close correlation between the flexion angle and the proportional alteration in capacitance. Similarly, the capacitance is gradually enhanced through a controlled flexion degree of the wrist, enabling precise recognition of distinct bending movements, as can be witnessed in Figure 5D. Hence, the proposed sensor has broad application prospects in sign language gesture recognition. All of this information can be gathered in order to create a database for sign language, which will greatly facilitate the online teaching of sign language, autonomous learning, and other applications. In conclusion, the proposed sensor exhibits precise responsiveness to various repetitive dynamic flexion and extension motions and show repeatable reactions and relaxation behaviors in each cycle. The flexible pressure sensor holds potential for functioning as a wearable sensor affixed to the skin, enabling the monitoring of human movements.

### Applications in vertical mechanical pressure

**FIGURE 6:**
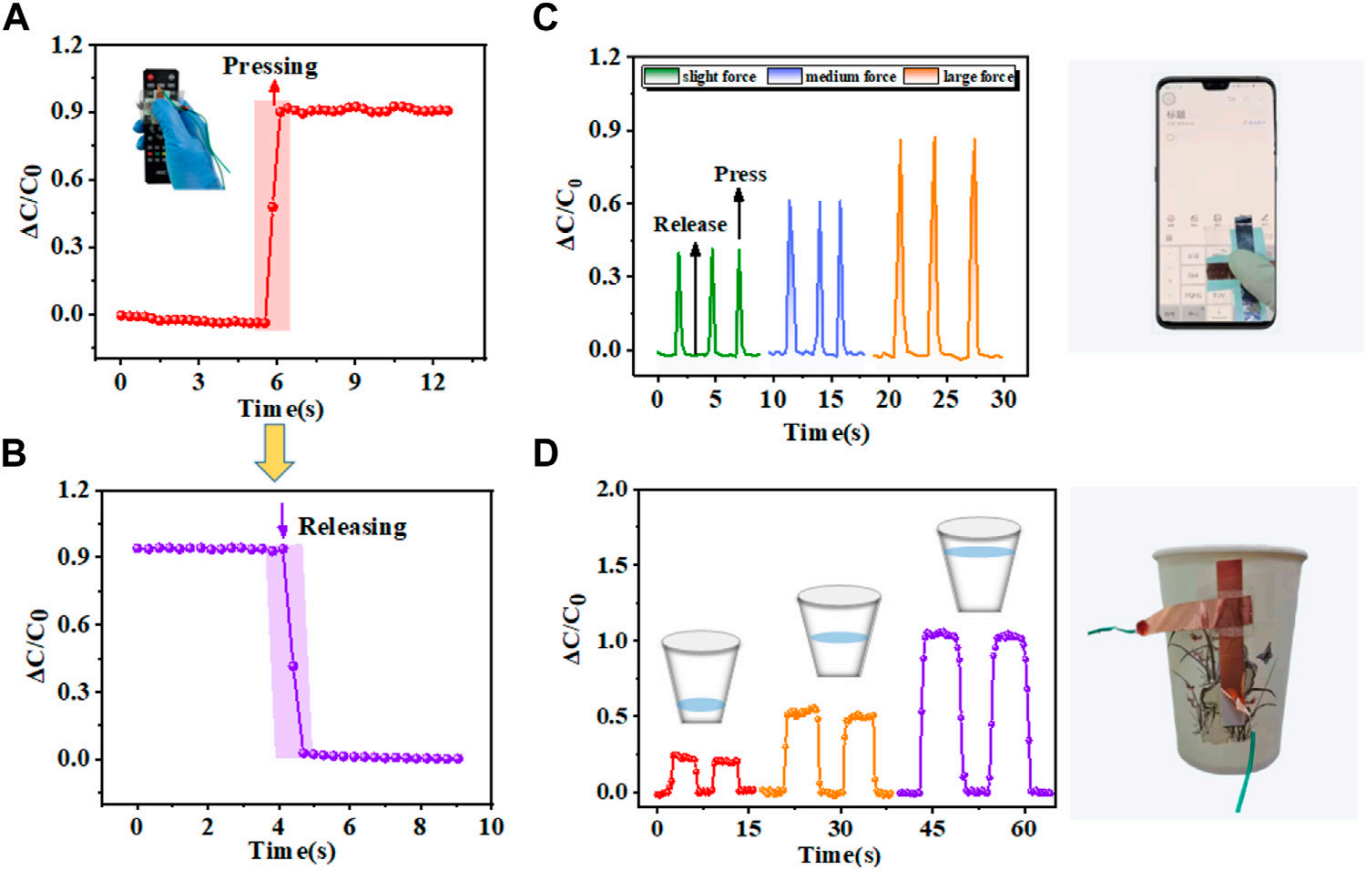
Applications in vertical mechanical pressure. **(A)** Capacitance response upon pressing the button on the remote control. **(B)** Capacitance response upon releasing the button on the remote control. **(C)** Variations in relative capacitance of sensor on phone screen pressed with different forces. **(D)** Variations in relative capacitance positioned on the lateral surface of the paper cup while handling paper cups of varying weights.

The pressure sensor also shows superior sensing performance in detecting vertical physical signals. The sensor was firstly mounted on the button of the remote control and constant continuous pressure is applied. The sensor’s response to pressure is illustrated in Figure 6A, showing its rapid and stable characteristics. Subsequently, upon force removal, Figure 6B demonstrates a prompt reduction in relative capacitance change. Furthermore, the sensor was attached to phone screen and Figure 6C displays the synchronous and stable capacitance curves as a result of applying three sets of distinct forces repeatedly on the sensor. Due to its excellent mechanical stability and remarkable sensitivity, this technology offers a promising solution for addressing touch screen malfunctions. Finally, the sensor was attached to the sidewall of the paper cup, which was filled with different volumes of water. The relative capacitance change is relatively small when grabbing the paper cup containing a small amount of water, as illustrated in Figure 6D. As the quantity of water increases, relative capacitance change will enlarge significantly. Besides, the relative capacitance alteration remains consistent during two iterations of grasping or releasing the paper cup. It is the special micro-pyramid structure of the pressure sensor that makes it has high sensitivity and good stability. These findings demonstrate that the suggested sensor can alleviate the human condition of tactile sensory disorder and also be applied to robot tactile perception (Chen et al., 2023; Liu et al., 2023; Zhen et al., 2023).

### Pressure mapping of sensor array

**FIGURE 7:**
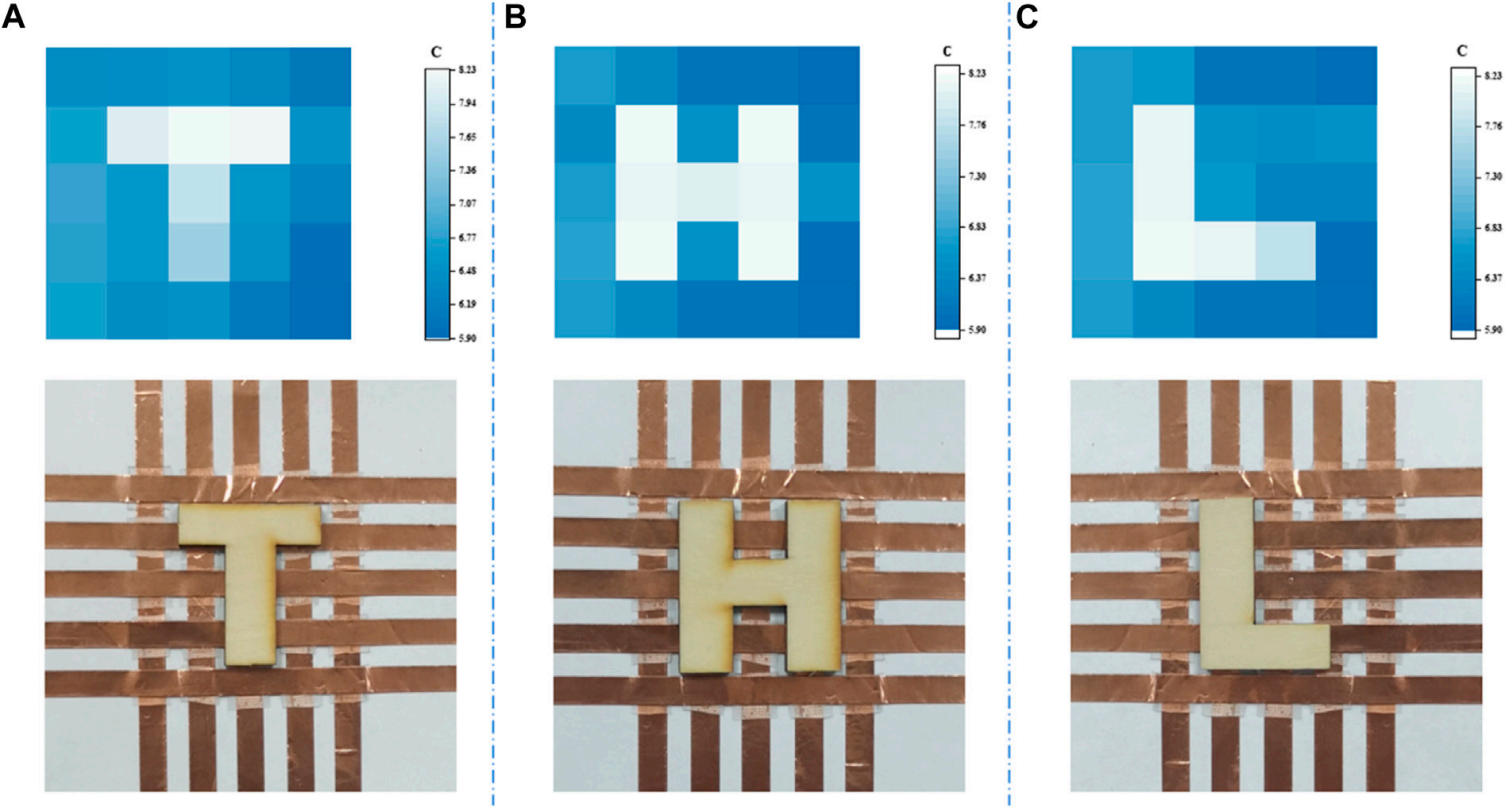
Spatial pressure allocation images of the 5 × 5 sensor array when loading wooden planks that are formed like the characters **(A)** “T”, **(B)** “H”, and **(C)** “L”.

A significant drawback of a solitary pressure sensor is its inherent limitation in furnishing comprehensive data. To enhance the applicability in practical scenarios, a 5 × 5 multipixel of the pressure sensor array was integrated to form a sensor array measuring 5 × 5 cm^2^, in which each pressure sensor measures 5 × 5 mm^2^. When the sensor array’s surface was covered by wooden boards bearing the shapes of “T", “H", and “L", their spatial distributions of the sensing response are shown in Figure 7. A noticeable contrast can be observed in the sensing response of the area that has been pressed and the one that has not. Meanwhile, the bright spot in the spatial pressure distribution image resembles the shape of a wooden board quite well, offering an efficient alternative in the wearable electronic device and flexible robot.

## Conclusion

In brief, an exceptionally sensitive and highly morphology-controllable flexible capacitive pressure sensor based on the hierarchical pyramid microstructure dielectric layer was successfully developed using an efficient strategy for pattern transfer of the silicon template. The hierarchically micro-pyramid structure’s distinctive design facilitates outstanding sensing performances through efficient stress concentration. The prepared flexible capacitive pressure sensor exhibits a remarkable sensitivity of 0.77 kPa^-1^ below 1 kPa. It also demonstrates an impressive response time of 55 ms and recovery time of 42 ms, along with a low detection limit of 8 Pa. Furthermore, this sensor showcases exceptional stability and reproducibility with up to 1,000 cycles. According to the findings from both experimental results and FEA simulations, it can be concluded that the sensing performance is significantly influenced by the thickness of the dielectric layer. The fabricated pressure sensor possesses the ability to continuously monitor pulse signals on the wrist and tracking human body movements in real-time. What is even more significant, an advanced 5 × 5 sensor array has been undertaken to demonstrate its capability in discerning different objects’ spatial pressure distribution. It is surely believed that the hierarchical pyramid microstructure by the silicon template-assisted manufacturing strategy can spark fresh ideas for advancing flexible pressure sensor technology and will pave the way for various applications including intelligent robotics, biomedical monitoring, smart prosthetics as well as disease prevention and diagnostics.

## Data Availability

The raw data supporting the conclusion of this article will be made available by the authors, without undue reservation.
